# Supplementary material for: Nomograms for Predicting Disease-Free Survival Based on Core Needle Biopsy and Surgical Specimens in Female Breast Cancer Patients with Non-Pathological Complete Response to Neoadjuvant Chemotherapy
Source: J Pers Med. 2023 Jan 29;13(2):249. doi: 10.3390/jpm13020249 (PMC9965597; doi:10.3390/jpm13020249)
Supplement: Supplementary file 1 [file jpm-13-00249-s001.zip › jpm-2141085-supplementary.pdf]

Journal of Personalized Medicine

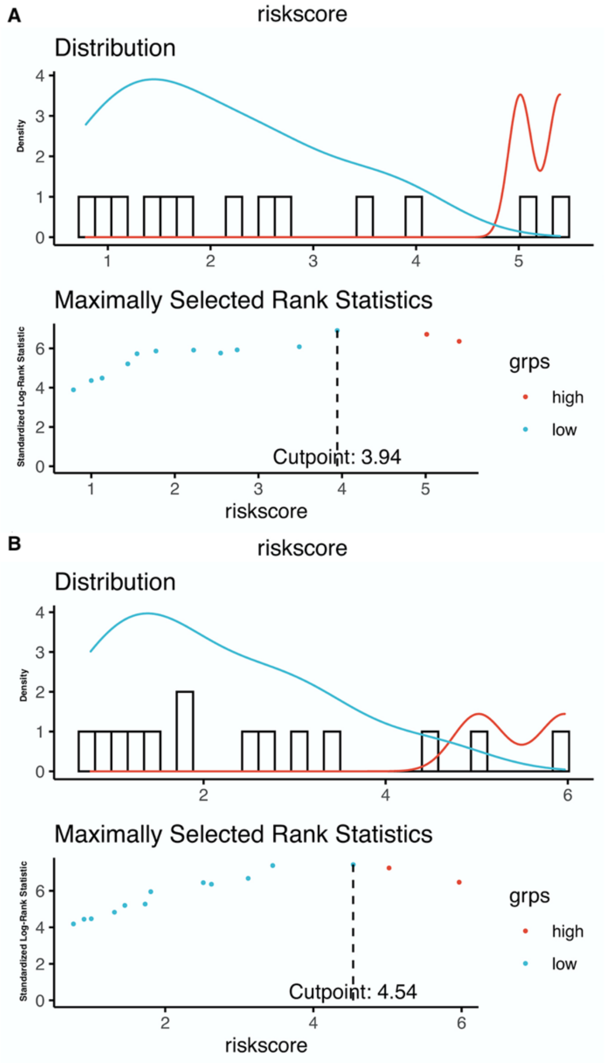

Supplementary Figure S1. The Maximally Selected Rank Statistics calculate cutpoint values of (A) the pre-NAC nomogram model and (B) the post-NAC model.

Abbreviations: NAC, neoadjuvant chemotherapy.

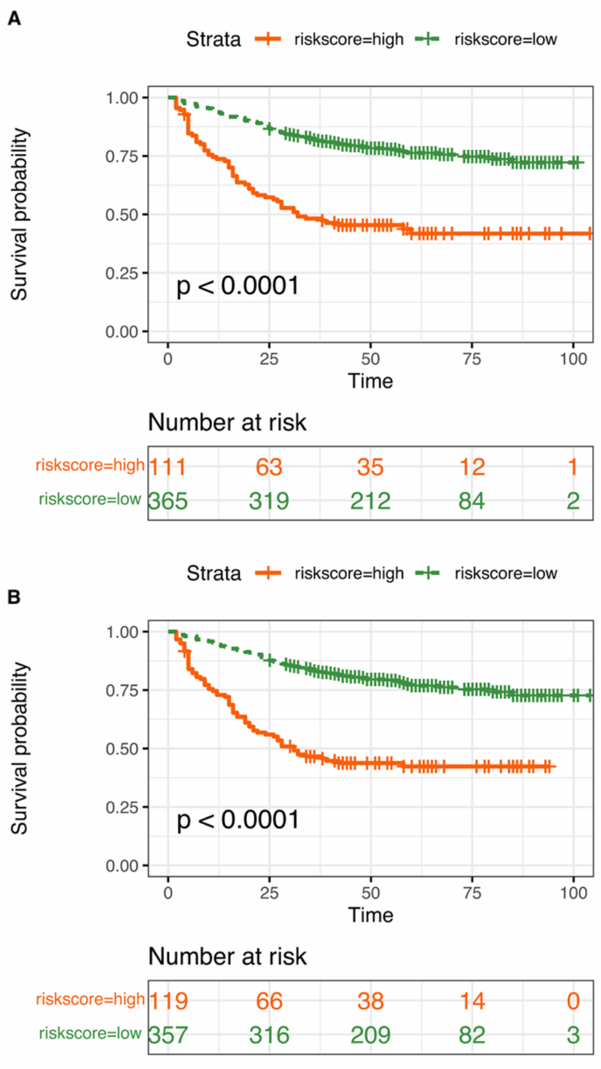

Supplementary Figure S2. DFS of patients in different risk subgroups in the primary cohort based on (A) pre-NAC nomogram model and (B) post-NAC nomogram model.

Abbreviations: DFS, disease-free survival; NAC, neoadjuvant chemotherapy.

Supplementary Table S1. Clinical characteristics of patients in the primary and validation cohorts.

Predictive factors	Primary cohort (*n* = 476) *n* (%)	Validation cohort (*n* = 131) *n* (%)	*p*
Age at diagnosis, y	49.0 (43.0-57.0)	49.0 (44.0-55.0)	0.948
Menopausal status			0.841
Pre-menopause	279 (58.6%)	75 (57.3%)	
Post-menopause	197 (41.4%)	56 (42.7%)	
cT			0.625
cT1	23 (4.8%)	4 (3.1%)	
cT2	333 (70.0%)	96 (73.3%)	
cT3+cT4	120 (25.2%)	31 (23.7%)	

cN			0.002
Negative	175 (36.8%)	29 (22.1%)	
Positive	301 (63.2%)	102 (77.9%)	
Pre-NAC ER status (%)			0.200
<45.0	242 (50.8%)	58 (44.3%)	
≥45.0	234 (49.2%)	73 (55.7%)	
Post-NAC ER status (%)			0.018
<32.5	252 (52.9%)	54 (41.2%)	
≥32.5	224 (47.1%)	77 (58.8%)	
Pre-NAC PR status (%)			1.000
<1.0	253 (53.2%)	69 (52.7%)	
≥1.0	223 (46.8%)	62 (47.3%)	
Post-NAC PR status (%)			0.250
<7.5	324 (68.1%)	82 (62.6%)	
≥7.5	152 (31.9%)	49 (37.4%)	
Pre-HER2 status			0.861
HER2-0	71 (14.9%)	21 (16.0%)	
HER2-low	223 (46.8%)	58 (44.3%)	
HER2-positive	182 (38.2%)	52 (39.7%)	
Post-HER2 status			0.393
HER2-0	72 (15.1%)	15 (11.5%)	
HER2-low	217 (45.6%)	57 (43.5%)	
HER2-positive	187 (39.3%)	59 (45.0%)	
Pre-NAC Ki67 status (%)			0.139
<22.5	250 (52.5%)	59 (45.0%)	
≥22.5	226 (47.5%)	72 (55.0%)	
Post-NAC Ki67 status (%)			0.485
<19.0	282 (59.2%)	73 (55.7%)	
≥19.0	194 (40.8%)	58 (44.3%)	
Pre-NAC p53 status (%)			0.074
<17.5	198 (41.6%)	66 (50.4%)	
≥17.5	278 (58.4%)	65 (49.6%)	
Post-NAC p53 status (%)			0.049
<17.5	233 (48.9%)	77 (58.8%)	
≥17.5	243 (51.1%)	54 (41.2%)	
Chemotherapy cycles			0.125
3	10 (2.1%)	1 (0.8%)	
4	432 (90.8%)	126 (96.2%)	
5-8	34 (7.1%)	4 (3.1%)	

cT clinical T staging, cN clinical nodal status, ER estrogen receptor, PR progesterone receptor, HER2 human epidermal growth factor receptor 2.

Supplementary Table S2. Association of risk change after NAC with DFS outcomes in the primary cohort.

Risk change	DFS events<br>*n* (%)	No DFS events<br>*n* (%)	Total<br>*n* (%)
Low-risk to low-risk	75 (22.2%)	263 (77.8%)	338 (71.0%)
High-risk to low-risk	5 (26.3%)	14 (73.7%)	19 (4.0%)
Low-risk to high-risk	10 (37.0%)	17 (63.0%)	27 (5.7%)
High-risk to high-risk	57 (62.0%)	35 (38.0%)	92 (19.3%)

NAC, neoadjuvant chemotherapy; DFS, disease-free survival; pCR, pathological complete response.
